# Clarifying contradictions: transportability in 17OHP-C trials and preterm birth outcomes using doubly debiased machine learning

**DOI:** 10.1093/aje/kwaf202

**Published:** 2025-09-24

**Authors:** Arti V Virkud, Eric Tchetgen Tchetgen, Enrique F Schisterman, Beth Pineles, Lisa D Levine, Stephen R Cole, Stefanie N Hinkle, Sunni Mumford, Kristin D Gerson, Brandie D Taylor, Sean Blackwell, Alan Peaceman, Samuel Parry, Mariah T Johnson, Dalynn Willis, Ellen C Caniglia

**Affiliations:** Department of Biostatistics, Epidemiology and Informatics, Perelman School of Medicine, University of Pennsylvania, Philadelphia, PA, United States; Department of Biostatistics, Epidemiology and Informatics, Perelman School of Medicine, University of Pennsylvania, Philadelphia, PA, United States; Department of Biostatistics, Epidemiology and Informatics, Perelman School of Medicine, University of Pennsylvania, Philadelphia, PA, United States; Department of Biostatistics, Epidemiology and Informatics, Perelman School of Medicine, University of Pennsylvania, Philadelphia, PA, United States; Department of Obstetrics and Gynecology, Perelman School of Medicine, University of Pennsylvania, Philadelphia PA, United States; Department of Obstetrics and Gynecology, Perelman School of Medicine, University of Pennsylvania, Philadelphia PA, United States; Department of Epidemiology, Gillings School of Public Health, University of North Carolina at Chapel Hill, Chapel Hill, NC, United States; Department of Biostatistics, Epidemiology and Informatics, Perelman School of Medicine, University of Pennsylvania, Philadelphia, PA, United States; Department of Biostatistics, Epidemiology and Informatics, Perelman School of Medicine, University of Pennsylvania, Philadelphia, PA, United States; Department of Obstetrics and Gynecology, Perelman School of Medicine, University of Pennsylvania, Philadelphia PA, United States; Department of Obstetrics and Gynecology, Perelman School of Medicine, University of Pennsylvania, Philadelphia PA, United States; Advocate Aurora Research Institute, Milwaukee, WI, United States; Department of Obstetrics, Gynecology, and Reproductive Sciences, UTHealth Houston McGovern Medical School, Houston, TX, United States; Obstetrics and Gynecology, Northwestern Feinberg School of Medicine, Chicago, IL, United States; Department of Obstetrics and Gynecology, Perelman School of Medicine, University of Pennsylvania, Philadelphia PA, United States; Department of Obstetrics and Gynecology, Hospital of the University of Pennsylvania, Philadelphia, PA, United States; PCORI Stakeholder Advisory Panel, Philadelphia, PA, United States; PCORI Stakeholder Advisory Panel, Philadelphia, PA, United States; Department of Biostatistics, Epidemiology and Informatics, Perelman School of Medicine, University of Pennsylvania, Philadelphia, PA, United States

**Keywords:** 17OHP-C, preterm birth, transportability, double debiased machine learning

## Abstract

Following the Meis et al. trial that identified a benefit of 17-alpha-hydroxyprogesterone caproate (17OHP-C) in reducing the risk of recurrent preterm birth (PTB) (risk difference (RD) −18.6%; 95% CI, −28.2%, −9.2%), a confirmatory trial (PROLONG) identified no benefit of 17OHP-C (RD, 1.2%; 95% CI, −3.0%, 5.3%). The leading hypothesis is that the difference was due to the heterogeneity in PTB risk. We implemented state-of-the-art methods, using doubly debiased machine learning for transportability to investigate whether the conflicting trial results could be explained by measured differences between trial populations. The estimated RD when transporting the effect in Meis to the PROLONG trial population was −18.6% (95% CI, −55.9%, 8.8%) comparing 17OHP-C to placebo. The estimated RD when transporting PROLONG to Meis was 5.2% (95% CI, −17.3%, 18.1%) comparing 17OHP-C to placebo. Transporting from PROLONG to Meis did not recover the protective effect observed in Meis, which we hypothesize is due to a hidden violation of one or more causal assumptions for transportability, such as the presence of unmeasured effect measure modifiers. Transporting from Meis to PROLONG did not recover the null point estimate observed in PROLONG, though the confidence interval was wide. Future studies should explore effect heterogeneity by PTB.

## Introduction

In 2003, a multi-center, large-scale randomized controlled trial conducted by Meis et al. identified a clinically significant protective effect of 17-alpha-hydroxyprogesterone caproate (17OHP-C) on the risk of recurrent preterm birth (PTB), defined as all delivery including spontaneous abortion and stillbirths less than 37 weeks, among women in the United States with a history of prior spontaneous PTB (risk difference (RD),18.6%; 95% CI, −28.2%, −9.2%). The Meis trial was halted early, and 17OHP-C received approval from the U.S. Food and Drug Administration (FDA) accelerated approval program, conditional on the results of a confirmatory trial.^1^ However, contrary to expectations, the subsequent confirmatory trial requested by the FDA (Progestin’s Role in Optimizing Neonatal Gestation, PROLONG) showed no effect of 17OHP-C in reducing the risk of recurrent PTB (RD, 1.2%; 95% CI, −3.0%, 5.3%), despite having an identical study protocol as Meis. The results of PROLONG, along with evidence from other prospective cohort studies, led the company that manufactures 17OHP-C to withdraw 17OHP-C from the market.^2^^-^^4^ The conflicting results from both trials have confused policymakers, clinicians, and their patients, particularly in the absence of approved alternative effective treatments to prevent recurrent PTB. This uncertainty has significant consequences, given that 10% of all births in the United States are preterm, and these births comprise the majority of perinatal morbidity and mortality with significant disparities across racial, ethnic, and socioeconomic groups.^5^^,^^6^

The leading hypothesis is that the discordant results in the 17OHP-C trials are due to heterogeneity in PTB risk between the two trial populations.^7^ While Meis was conducted solely in the United States, PROLONG recruited at U.S. and non-U.S. sites (mainly in Eastern Europe). By the time PROLONG recruitment was initiated in 2009, 17OHP-C had already been widely adopted in U.S. clinical care, limiting the enrollment of participants in the United States.^7^ Compared to participants in PROLONG, individuals enrolled in Meis had a greater number of prior PTBs, a younger gestational age for the qualifying PTB, higher body mass index (BMI), fewer years of education, were more likely to use substances during pregnancy, and were more likely to identify as non-Hispanic Black (59% in Meis vs 7% in PROLONG).

Other possible explanations for the discordant results include differences in protocol deviations between the trials due to loss to follow-up, noncompliance or unblinding, trial outcome definitions, measurement error of the outcome, the distribution of multiple versions of treatment, type I error, and/or imbalance of baseline risk factors of PTB. However, both Meis and PROLONG trials had minimal loss to follow-up and noncompliance, used the same gold-standard measurement for the outcome, and examined a well-defined pharmaceutical treatment with identical formulations, making many of these explanations for conflicting results unlikely. The CIs around the point estimates also make the likelihood of type I error small. Here, we investigate whether between-study differences in observed measured baseline characteristics, considered as potential effect measure modifiers of 17OHP-C, could explain the conflicting results of the trials of 17OHP-C and PTB.

While randomized clinical trials with identical study protocols sometimes do produce conflicting results, there is no consensus on a formal analytic approach for reconciling such conflicts. Methods for transportability have been developed that, under certain assumptions, can estimate the causal effect of the treatment of interest in a target population that may be distinct from the study sample.^8^ This allows investigators to *transport* the estimated effect in a study population to a separate population representing a target population.

Consider the hypothesis that the differences in study results are solely attributable to variations in the distribution of effect measure modifiers between the studies. In this scenario, participants in the Meis study were observed to be at higher risk for PTB than those in the PROLONG study due to measured risk factors (such as the number of previous PTBs and gestational age at qualifying PTB), and the beneficial effect of 17OHP-C observed in Meis could be explained by increased efficacy of treatment in a higher-risk population—one that had a greater baseline likelihood of PTB and therefore a higher potential for risk reduction. Conversely, in PROLONG, where the study population had a lower overall risk profile, the same treatment effect might not have been observed because the absolute risk reduction was smaller or negligible. This would suggest that the effectiveness of 17OHP-C is contingent upon the underlying risk profile of the population, highlighting the importance of effect modification in interpreting these results. In this hypothetical, transporting effect estimates from Meis to PROLONG should recover a null effect of 17OHP-C on PTB within the PROLONG population, and transporting estimates from PROLONG to Meis should generate a protective effect of 17OHP-C on PTB within the Meis population.

Here, we adapt existing transportability methodology to reconcile the differences between Meis and PROLONG. The original method was designed to transport from a trial in which an intervention was evaluated to a target population of trial-eligible individuals. In contrast, because the same intervention was evaluated in both trial populations, our approach transports each trial to the other to evaluate whether effect measure modifiers captured in the baseline characteristics explain the difference in observed effects between the two trials. Additionally, we enhance existing statistical methods that overly rely on parametric model specifications by leveraging modern machine learning methods to flexibly and data adaptively estimate regression models needed to transport estimated effects.

## Methods

### Data source and study population

We obtained deidentified clinical data from both trials from AMAG Pharmaceuticals, Inc. Meis (2003) was conducted among 463 pregnant participants in the United States; the trial was stopped early due to the observed benefit after reaching 92.6% of the recruitment target.^1^ PROLONG (2020) included 1708 pregnant participants across 93 centers in 9 countries.^2^ Both trials were randomized, placebo-controlled, double-blind studies comparing 17OHP-C to placebo for pregnant individuals with at least one prior spontaneous PTB. In each trial, pregnant participants were randomized to receive a weekly injection of 17OHP-C or placebo from enrollment between 16 weeks and 20 weeks and 6 days of gestation until delivery. Both trials assessed multiple efficacy and safety outcomes.

In Meis, the primary outcome was delivery at less than 37 weeks of gestation based on delivery records, which included stillbirths, miscarriages, and abortions. PROLONG had two primary efficacy outcomes, including delivery at less than 35 weeks of gestation and a composite neonatal morbidity and mortality index. PROLONG evaluated delivery at less than 37 weeks of gestation as a secondary outcome.

### Statistical analysis

After replicating the frequencies of the measured baseline characteristics from the original trials and comparing the populations of both studies, we first estimated unadjusted and adjusted risk differences for preterm delivery by randomization arm, and corresponding 95% CIs derived from the nonparametric bootstrap (10 000 bootstrap samples).^9^ Adjusted risk differences of the original trials were estimated using inverse probability of treatment weights modeled with a parametric logistic regression model that accounted for all baseline characteristics.^10^ We estimated adjusted risk differences because we observed an imbalance between treatment arms in the number of prior preterm deliveries, smoking behavior during pregnancy, substance use during pregnancy, and alcohol use during pregnancy. Additionally, we adjusted for baseline characteristics such as gestational age (in weeks) at delivery in the qualifying prior PTB, maternal age, race/ethnicity, marital status, pre-pregnancy maternal BMI, and years of education. The qualifying prior PTB was the PTB that occurred most recently prior to the trial and qualified the pregnant woman to enter the trial. Gestational age at randomization was roughly identical across trials and was not included in adjustment. Additional details on coding of baseline characteristics and missing value treatment are included in the Appendix (Appendix S1).

We then used our adapted transportability methodology to transport the intention-to-treat effect of assignment to 17OHP-C vs placebo on PTB from Meis to PROLONG and from PROLONG to Meis.^8^ The availability of effect estimates in both trial populations is unique for transportability questions because we can directly compare the transported estimate to the observed effect in the trial population to which we transported, as an empirical test of the hypothesis that differences in the distribution of measured effect modifiers between the two trials explain the conflicting trial results (ie, that all relevant effect measure modifiers have been measured accurately).

We define the potential outcome ${Y}^a$, as an individual’s occurrence of a recurrent PTB had they, possibly contrary to fact, received treatment *a*, where *a* = 1 encodes receiving 17OHP-C injection, while *a* = 0 encodes receiving placebo. We also let *A* denote randomized treatment assignment, *X* represent measured baseline covariates, and *S* encode trial participation.

The causal identifiability assumptions for transportability from Meis to PROLONG include: (1) conditional exchangeability in the mean over *A* (where Meis measured baseline confounders are balanced between the 17OHP-C and placebo arms in Meis), (2) consistency of potential outcomes, (3) positivity of 17OHP-C assignment in Meis, (4) conditional exchangeability in the mean over *S* (where measured effect measure modifiers are balanced between Meis and PROLONG), and (5) positivity of trial participation in Meis. The assumptions necessary for transport from PROLONG to Meis are analogous. Assumptions 1-3 are expected to hold for well-defined interventions assessed in randomized trials. Assumption 5 (positivity assumption) was empirically evaluated by examining cell sizes to diagnose violations. If the transported and observed effects differ beyond sampling variability, this difference could potentially be due to violation of assumption 4 (the presence of unmeasured effect measure modifiers).

Given these causal assumptions, the transported causal effect from Meis to the PROLONG study population is given by the following formula:


\begin{align*} {\mu}_{\mathrm{MtoP}}&=E\big[E\left[Y|X,S=\mathrm{Meis},A=1\right]-E\left[Y|X,S=\mathrm{Meis},A=0\right]|\\S&=\mathrm{PROLONG}\big]. \end{align*}

To transport the causal effect from PROLONG to the Meis study population, the estimand of interest is analogous:


\begin{align*} {\mu}_{\mathrm{PtoM}}&=E\big[E\left[Y|X,S=\mathrm{PROLONG},A=1\right]-E\big[Y|X,\\S&=\mathrm{PROLONG},A=0\big]|S=\mathrm{Meis}\big]. \end{align*}

Because of the observed imbalance in baseline covariates, we compare the transported effects to the adjusted effects rather than the unadjusted effects observed in both trials. We are evaluating the mathematical hypotheses as an immediate consequence of the hypothesis that *X* captures all relevant effect modifiers (on the additive scale):


(1) \begin{equation*} {H}_0^1:E\left\{{Y}^{a=1}-{Y}^{a=0}|S=\mathrm{PROLONG}\right\}={\mu}_{\mathrm{MtoP}} \end{equation*}

(2) \begin{equation*} {H}_0^2:E\left\{{Y}^{a=1}-{Y}^{a=0}|S=\mathrm{Meis}\right\}={\mu}_{\mathrm{PtoM}}. \end{equation*}

Conceptually, the hypothesis that one has accurately measured all relevant effect measure modifiers implies ${H}_0^1$ and ${H}_0^2$. Therefore, evaluating ${H}_0^1$ and ${H}_0^2$ would provide circumstantial evidence to reject the hypothesis that all relevant effect measure modifiers have been measured. However, failing to reject ${H}_0^1$ and ${H}_0^2$ does not necessarily imply that all relevant effect measure modifiers have been measured, but rather suggests that evidence to the contrary may be challenging to obtain given the observed data.

To evaluate the transported effect, we implemented a doubly debiased machine learning (DDML) estimator to obtain all primary results for ${\mu}_{\mathrm{MtoP}}$ and ${\mu}_{\mathrm{PtoM}}$.^8^^,^^11^ The advantage of this estimator is that it does not rely on parametric assumptions to estimate all required regression models. Our DDML consists of a doubly robust estimator from the transportability literature,^8^ SuperLearner as the ensemble learner to fit nuisance regression functions, and cross-fitting of nuisance functions.^11^^,^^12^ The relevant nuisance functions for the doubly robust estimator include the propensity score (probability of treatment *a* given covariates*,*  $\Pr \left[A=a|X=x,S=s\right]\big)$ in Meis and PROLONG, the probability of trial participation given covariates ($\Pr \left[S=s|X=x\right]\big)$, and the outcome regression for standardization $E\left(Y|A,S,X\right)$.^12^ In our implementation of SuperLearner, we included generalized linear models, generalized additive models, gradient boosted machines, random forests, XGBoost, and neural nets.^13^^-^^17^

We then calculated the difference between the transported effect and the observed effect for both trials: $E\big\{{Y}^{a=1}-{Y}^{a=0}|S=\mathrm{PROLONG}\big\}-{\mu}_{\mathrm{MtoP}}$ and $E\left\{{Y}^{a=1}-{Y}^{a=0}|S=\mathrm{Meis}\right\}-{\mu}_{\mathrm{PtoM}}$. Confidence intervals were calculated using nonparametric bootstrap (10 000 bootstrap samples).^9^ In each bootstrap, we re-estimated the adjusted causal risk difference in the full sample to generate a Wald confidence interval for the difference between the two effects.

We conducted several subgroup analyses. We restricted the PROLONG study to U.S. participants only and transported the causal effect from PROLONG-U.S. to the Meis study population and transported the causal effect from Meis to the PROLONG-U.S. study population. By restricting the population to U.S. participants, unmeasured modifiers associated with the region may be partially adjusted. We also conducted subgroup analyses among trial participants who (1) had experienced only one prior PTB; (2) did not smoke, drink alcohol, or use substances during pregnancy; (3) had smoked, drank alcohol, or used substances during pregnancy; (4) had previously experienced preterm premature rupture of membranes; and (5) had not previously experienced preterm premature rupture of membranes. The subgroups were identified as factors that, either directly or indirectly, may contribute to the effect heterogeneity of 17OHP-C on PTB.

We also completed a series of sensitivity analyses. We transported the causal effects using direct standardization via outcome regression, normalized inverse odds weights, and parametric doubly robust estimator separately.^8^ While the DDML estimator is the primary result, observing differences between the results for the parametric components (standardization via outcome regression and normalized inverse odds weighting) provided additional understanding of model misspecification. To investigate the potential impact of near violations of the positivity of trial participation assumption (assumption 5), we restricted the original trial, from which the weights are generated, to the population of individuals where the probability of being in the trial given baseline covariates was greater than 0.1. Finally, we evaluated two additional outcomes: spontaneous PTB and premature rupture of membranes PTB. All analyses were conducted in R (version 2024.09.0).^18^ This study was determined to be exempt by the Institutional Review Board at the University of Pennsylvania.

## Results

We replicated the original descriptive statistics from each trial (Table 1). We also replicated the primary results from each trial (Table 2). The unadjusted risk difference (and corresponding 95% CI in parentheses) for PTB comparing 17OHP-C to placebo was −18.6% (−28.2%, −9.2%) in Meis and 1.2% (−3.0%, 5.3%) in PROLONG. The adjusted risk difference for PTB comparing 17OHP-C to placebo was −17.5% (−27.2%, −7.8%) in Meis and 1.0% (−3.1%, 4.8%) in PROLONG.

**Table 1:** Comparison of baseline characteristics in the Meis and PROLONG trials of 17OHP-C and PTB.

	**Meis**		**PROLONG**	
**Characteristic**	**17OHP-C (*n* = 310)**	**Placebo (*n* = 153)**	**17OHP-C (*n* = 1130)**	**Placebo (*n* = 578)**
Gestational age at qualifying prior PTB (week)^a^	30.6 ± 4.6	31.3 ± 4.2	31.3 ± 4.4	31.6 ± 4.2
>1 previous preterm delivery—no. (%)	86 (27.7)	63 (41.2)	148 (13.1)	70 (12.1)
Age (years)^a^	26.0 ± 5.6	26.5 ± 5.4	30.0 ± 5.2	29.9 ± 5.2
Race or ethnic group, no. (%)^b^				
Asian	2 (0.6)	1 (0.7)	23 (2.0)	22 (3.8)
Hispanic	43 (13.9)	26 (17.0)	101 (8.9)	54 (9.3)
Non-Hispanic Black	183 (59.0)	90 (58.8)	73 (6.5)	41 (7.1)
Non-Hispanic White	79 (25.5)	34 (22.2)	1004 (88.8)	504 (87.2)
Other	3 (1.0)	2 (1.3)	30 (2.7)	11 (1.9)
Married or living with partner, no. (%)	159 (51.3)	71 (46.4)	1013 (89.6)	522 (90.3)
Prepregnancy BMI (kg/m^2^)^a^	26.9 ± 7.9	26.0 ± 7.0	24.3 ± 7.0	24.7 ± 8.6
Years of education^a^	11.7 ± 2.3	11.9 ± 2.3	13.0 ± 2.4	13.0 ± 2.4
Smoking during pregnancy, no. %	70 (22.6)	30 (19.6)	92 (8.1)	41 (7.1)
Alcohol use during pregnancy, no. %	27 (8.7)	10 (6.5)	24 (2.1)	18 (3.1)
Substance use during pregnancy, no. %	11 (3.5)	4 (2.6)	16 (1.4)	8 (1.4)

a Data expressed as mean ± SD.

b Race and ethnicity measured separately in PROLONG, so each variable was treated mutually exclusively in paper.

**Table 2:** Unadjusted/adjusted absolute risks, risk differences, and risk ratios for PTB comparing 17OHP-C to placebo in the trials of 17OHP-C and PTB.

**Absolute risk (unadjusted)**	**Meis (2003)**	**PROLONG (2020)**
17OHP-C	36.3%	23.1%
Placebo	54.9%	21.9%
Absolute risk (adjusted)	Meis (2003)	PROLONG (2020)
17OHP-C	36.8%	22.9%
Placebo	54.3%	21.9%
Risk differences	Meis (2003)	PROLONG (2020)
Unadjusted	−18.6% (−28.2, −9.2)	1.2% (−3.0, 5.3)
Adjusted^a^	−17.5% (−27.2, −7.8)	1.0% (−3.1, 4.8)
Risk ratios	Meis (2003)	PROLONG (2020)
Unadjusted	0.66 (0.54, 0.81)	1.06 (0.88, 1.28)
Adjusted^a^	0.68 (0.55, 0.84)	1.04 (0.87, 1.26)

a Adjusted for gestational age (in weeks) at delivery in the qualifying previous PTB, number of previous preterm deliveries, maternal age, race/ethnicity, marital status, pre-pregnancy maternal BMI, years of education, smoking status during pregnancy, alcohol use during pregnancy, and substance use during pregnancy.

We evaluated the theoretical validity of the causal identifiability conditions required for a causal interpretation of the aforementioned estimands. Because this analysis involved two randomized controlled clinical trials of well-defined interventions, we did not expect violations of the consistency of potential outcomes or positivity of 17OHP-C assignment (assumptions 2 and 3). However, the proportion of individuals with more than one previous preterm delivery was imbalanced across treatment arms in the Meis trial (27.7% in 17OHP-C vs 41.2% in placebo). While not a direct violation of the conditional exchangeability assumption (assumption 1), one might theorize that an imbalance of measured covariates could indicate an imbalance of *unmeasured* risk factors for PTB. In investigating the propensity of selection in each trial given the covariates (assumption 5), we confirmed there were no estimated probabilities close to zero. As described above, by comparing the estimates obtained via transportability to the observed effects in the trial to which we transport, we are empirically evaluating assumption 4 that all relevant effect measure modifiers are measured.

**Figure 1:**
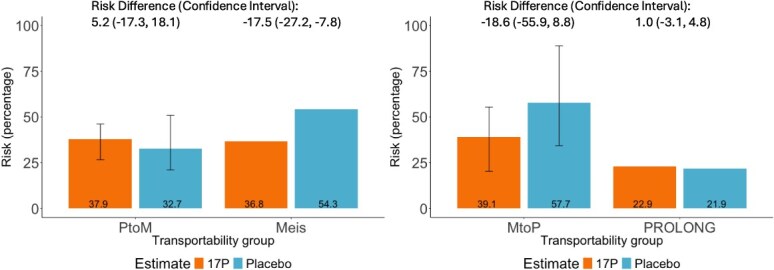
Comparing DDML results (PROLONG, *n* = 1684; Meis, *n* = 459) with adjusted study estimates. Transportation group results are as follows: PtoM represents PROLONG transported to the Meis population. Meis represents adjusted results from the original Meis 2003 study. MtoP represents Meis transported to the PROLONG population. Prolong represents adjusted results from the original PROLONG 2020 study.

Figure 1 shows the adjusted absolute risks and risk differences obtained in the original trials and those obtained after transporting estimates from Meis to PROLONG and PROLONG to Meis using DDML (results from all estimators are shown in Tables S1-S2). The estimated risk difference transporting Meis to PROLONG was −18.6% (95% CI, −55.9%, 8.8%) comparing 17OHP-C to placebo. The estimated risk difference transporting PROLONG to Meis was 5.2% (95% CI, −17.3%, 18.1%) comparing 17OHP-C to placebo. The difference between the adjusted risk difference in PROLONG and the Meis-to-PROLONG transported effect was 19.6% (95% CI, −8.1%, 57.3%). The difference between the adjusted risk difference in PROLONG and the Meis-to-PROLONG transported effect was 22.7% (95% CI, −37.9%, 2.0%).

**Figure 2:**
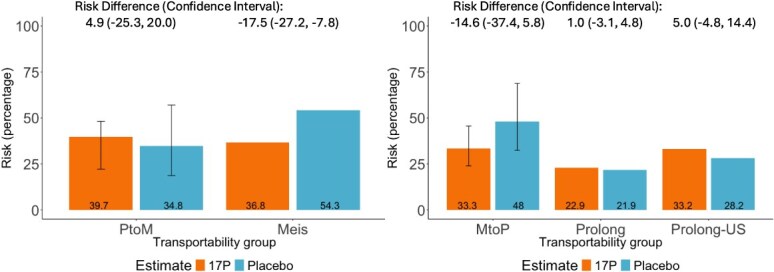
Comparing DDML results among U.S. trial participants (PROLONG, *n* = 387; Meis, *n* = 459) with adjusted study estimates. Transportation group results are as follows: PtoM represents U.S. PROLONG transported to the Meis population. Meis represents adjusted results from the original Meis 2003 study. MtoP represents Meis transported to the U.S. PROLONG population. Prolong represents adjusted results from the original PROLONG 2020 study. Prolong-U.S. represents the U.S. trial participants in the original PROLONG 2020 study.

**Figure 3:**
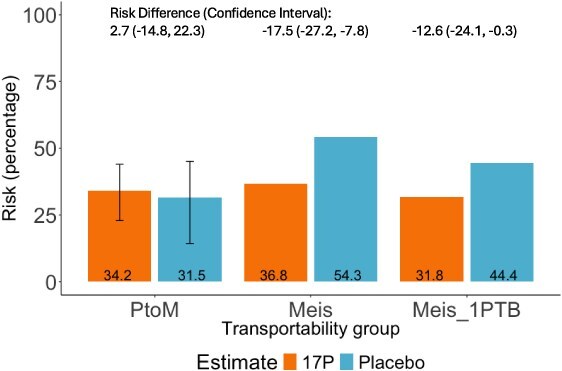
Comparing DDML results among trial participants with only one prior PTB (PROLONG, *n* = 1468; Meis, *n* = 310) with adjusted study estimates. Transportation group results are as follows: PtoM represents PROLONG participants with only one prior PTB transported to the Meis population with only one prior PTB. Meis represents adjusted results from the original Meis 2003 study. Meis_1PTB represents the trial participants with only one prior PTB in the original Meis 2003 study.

Replicating this transportability approach in a U.S.-only trial population and among participants with only 1 prior PTB generated similar results to the primary analysis (Figures 2 and 3 and Tables S3-S4). We were unable to use SuperLearner to estimate the DDML result when transporting Meis to PROLONG among trial participants with only 1 prior PTB, because the sample size was too small (*n* = 310). The doubly robust estimate of transporting Meis to PROLONG among U.S. participants was attenuated (−14.6%, 95% CI, −36.7%, 4.8%).

The remaining subgroup and sensitivity analyses did not produce substantively different results, with a few exceptions (Tables S7-S20). Due to sample size, we were unable to generate estimates for select estimators within certain subgroup and sensitivity analyses (Tables S5, S6, S10, S11, S12, S14). When transporting from Meis to PROLONG for the outcome of spontaneous PTB (Appendix S3), the DDML point estimate was closer to the null effect observed in PROLONG (RD, −3.6%; 95% CI, −42.5%, 20.7%, Table S18), but this was not mirrored when transporting from PROLONG to Meis (5.3%, 95% CI, −12.6%, 20.8%; Table S17). There was greater reconciliation in both directions when examining the outcome of preterm premature rupture of membranes deliveries (PROLONG to Meis RD, −1.9%; 95% CI, −16.1%, 11.5% and Meis to PROLONG RD, −1.4%; 95% CI, −14.3%, 10.9%; Tables S19 and S20).

## Discussion

We adapted existing transportability methodology to attempt to reconcile the conflicting results from the Meis and PROLONG trials. Although there were several large observed differences in characteristics between the two trials, we were not able to recover the protective effect observed in Meis by transporting the causal effect from PROLONG to the Meis trial population. When transporting the causal effect from Meis to the PROLONG trial population, the DDML estimator yielded a large protective point estimate (in contrast to the PROLONG study’s approximately null point estimate) but a sufficiently high uncertainty (a wide 95% CI containing the null) to encompass the scenario of recovering the causal effect observed in PROLONG. While the parametric outcome model estimator had comparable variability to the DDML estimator, the parametric inverse odds weighted and doubly robust estimators had less variability (95% CIs excluded the null, which was inconsistent with recovering the effect in PROLONG). The number of covariates adjusted for in the nuisance functions and the smaller sample size of Meis may contribute to why the DDML and parametric outcome model yielded imprecise estimates. However, as described above, the corresponding PROLONG to Meis transported estimates did not reconcile the results. Reconciliation in both directions is necessary to conclude we completely reconciled the results.

The sensitivity analyses were designed to explore if slight modifications to the study population could aid in reconciling the conflicting results from the Meis and PROLONG trials. Transporting to and from only the PROLONG-U.S. population addressed possible unmeasured effect measure modifiers distinguishing the U.S. and non-U.S. populations. Applying transportability methods to only those individuals with a probability of being selected into the trial given the participant’s covariates greater than 0.1 (P(S|X) > 0.1) helped to address residual confounding and/or effect measure modification among participants who had a lower likelihood of being selected in either trial. We conducted additional subgroup analyses to account for additional effect heterogeneity introduced by each subgroup. While examining preterm premature rupture of membrane delivery as an outcome, there seemed to be some reconciliations, suggesting that the heterogeneity in the outcome of PTB could partially explain the conflicting results.

While we evaluated the circumstantial evidence of each of the causal identifiability assumptions before conducting this analysis, these assumptions cannot be empirically evaluated. Since our analysis compared two randomized trials, we did not expect there to be violations of consistency of potential outcomes, particularly given the identical protocols in the administration of 17OHP-C injections. For a similar reason, we do not expect a violation of the positivity of treatment assignment. We hypothesize that violations of some of the other causal identifiability assumptions may be responsible for the observed results.

First, there is the possibility of bias introduced by unmeasured effect measure modification. The two trial study populations have clear differences, as evidenced by the risk of PTB in the placebo arms and the descriptive statistics of baseline participant characteristics mentioned above. All effect measure modifiers must be measured to achieve conditional exchangeability in mean over S. It is possible that the measured characteristics are not the full set of effect measure modifiers relevant to the effect of 17OHP-C on preterm delivery. Some unmeasured effect measure modifiers could include access to and utilization of quality care, experiences of racism and discrimination, and changes in care and 17OHP-C availability due to the different eras the trials were conducted in that are likely to vary substantially between the two study populations.

Second, there is the potential for chance imbalance in the Meis trial that would prevent reconciliation using this transportability approach. A greater percentage of participants randomized to the placebo group versus 17OHP-C group had previously experienced more than 1 preterm delivery (41.2% vs 27.7%). While both trials can be subject to chance imbalance, the evidence of imbalance by key measured variables within Meis raises concern that other imbalances (across unmeasured variables not stratified in the trial design) could also be present in the trial by chance. We can use the Vanderweele bias equation, a calculation for the maximum amount by which an unmeasured confounder could reduce an observed risk ratio, to evaluate the impact of an unmeasured confounder.^19^ If an unmeasured confounder were similarly associated with treatment arm (risk ratio (RR_EU_) of 1.5, where E is 17OHP-C and U is a potential unmeasured confounder) and had a modest or strong association with PTB, it is possible that this unmeasured confounding could explain the differences in results between the two trials. For example, cervical length was unmeasured in Meis and cervical lengths at or below the 25th percentile have been shown to increase the risk of PTB by as much as 3-fold (RR_UD_, where D is PTB).^20^ If short cervix was similarly imbalanced in Meis, the adjusted risk ratio would shift to 0.84 with a 95% CI ranging from 0.69 to 1.04 (using the Vanderweele bias equation RR_UD_*RR_EU_/[RR_UD_ + RR_EU_ − 1]).^19^

A combination of unmeasured baseline covariate imbalances and unmeasured effect measure modification might explain our inability to reconcile the conflicting results. Finally, even if the identifiability assumptions were all met, it is still possible that we were not able to reconcile the conflicting results due to other differences between the two trials, like protocol deviations leading to postbaseline confounding.

While PROLONG was designed to replicate the Meis study protocol, the two trials were different. PROLONG was an international, multicenter trial conducted across 9 countries, whereas Meis was conducted at sites exclusively in the United States. PROLONG was restricted to pregnant participants aged 18 years or older, whereas Meis recruited participants aged 16 or older and had a generally younger cohort. Meis had a higher percentage of participants of color, participants with more than one previous preterm delivery, participants who were divorced/single, participants with a higher pre-pregnancy BMI, and participants who had smoked or used alcohol during pregnancy. All together, these baseline characteristics describe the differences that may explain the higher risk of PTB observed in the placebo arm in Meis (54.9%) vs PROLONG (21.9%). While the inclusion criteria restricted trial participants to individuals who had specifically experienced a spontaneous PTB, the outcome included all types of preterm deliveries. Preterm delivery is a heterogeneous condition, making it reasonable to expect that certain biological characteristics could influence the benefit of 17OHP-C in preventing PTB. However, this transportability analysis did not identify any specific differences between study participants in these two trials that explain the observed differences in the effect of 17OHP-C on PTB.

Several future studies are suggested based on the results of this work. While select violations of the causal identifiability assumptions may not be feasibly confirmed with the trial data alone, alternative methods can be explored to address possible violations. The use of SuperLearner to estimate the propensity score relaxed some of the parametric assumptions in the nuisance parameters and estimation procedures, but the transportability estimation included assumptions on the data distribution that could be relaxed in a nonparametric estimation of the conditional average treatments.^21^ By exploring individual treatment rules and grouped average treatment effects, we can explore more complex effect-measure modification relationships to ascertain if certain subgroups of patients experience more or less of a protective effect.^11^^,^^22^

In this paper, we developed an approach that advances existing transportability methods by leveraging machine learning to relax parametric model assumptions. While we are still uncertain why the two trials produce different evidence on the effect of 17OHP-C, this analysis suggests the difference is not due to the measured modifiers in this study. The discrepancy may be due to biological, genetic, or environmental effect measure modifiers unmeasured in these trials. Future studies can collect data on these unmeasured characteristics to uncover potential heterogeneity among pregnant persons who have experienced a PTB.

## Supplementary Material

Web_Material_kwaf202

## Data Availability

The data in this publication are available through COVIS.
