# Supplementary material for: Clarifying contradictions: transportability in 17OHP-C trials and preterm birth outcomes using doubly debiased machine learning
Source: Am J Epidemiol. 2025 Sep 24;195(8):2237–43. doi: 10.1093/aje/kwaf202 (PMC13437081; doi:10.1093/aje/kwaf202)
Supplement: Supplementary file 1 [file web_material_kwaf202.zip › Supplemental_FIRSTLOOKFINAL.docx]

***American Journal of Epidemiology* Submitted Manuscript**

**Special Collection:** N/A

**Title:** Clarifying Contradictions: Transportability in 17OHP-C Trials and Preterm Birth Outcomes Using Doubly Debiased Machine Learning

**Authors:** Arti V. Virkud, Eric Tchetgen Tchetgen, Enrique F. Schisterman, Beth Pineles, Lisa D. Levine, Stephen R. Cole, Stefanie N. Hinkle, Sunni Mumford, Kristin D. Gerson, Brandie D. Taylor, Sean Blackwell, Alan Peaceman, Samuel Parry, Maria T. Johnson, Dalynn Willis, Ellen C. Caniglia

List: Appendices S1, S2, S3, Tables S1-S20

Appendix

S1. Details on baseline characteristics

We harmonized select baseline characteristics to compare the two studies and implement the transportability methodology. In Meis, the trial measured marital status using prenatal records as a 3-level covariate, with being “divorced, widowed, or separated” treated as a separate category, whereas the PROLONG trial classified “divorced, widowed, or separated” individuals, if present in the trial sample, as single. We reclassified Meis participants identifying as this third separate marital status as single. The PROLONG trial assessed race and ethnicity separately, however, Meis combined these identifiers for individuals self-assigning as “Asian” or “Other”. Therefore, we were restricted to classifying race/ethnicity according to the Meis trial.

We also addressed missing values in 20 participants by employing the missing data indicator approach for the following covariates: gestational age, number of prior preterm deliveries, pre-pregnancy BMI, and/or years of education.^1,2^ Missing values were set to the median for continuous and count variables and the reference level for categorical variables.

S2. Results from subgroup and sensitivity analyses

**Table S1 PROLONG (n=1684) transported to Meis (n=459)**

| Estimator | 17P | Placebo | Risk Difference | Risk Ratio |
| --- | --- | --- | --- | --- |
| Meis* | 36.8% | 54.3% | -17.5% (-27.7, -8.2) | 0.68 (0.55, 0.84) |
| OM | 36.7% (29.3, 44.6) | 34.5% (25.1, 45.7) | 2.2% (-10.7, 14.7) | 1.06 (0.74, 1.57) |
| IOW | 38.9% (27.9, 51.2) | 36.6% (24.0, 52.2) | 2.3% (-16.1, 19.7) | 1.06 (0.66, 1.77) |
| DR | 39.8% (30.5, 51.0) | 35.3% (23.1, 48.0) | 4.5% (-10.8, 21.3) | 1.13 (0.75, 1.89) |
| DDML | 37.9% (26.4, 45.8) | 32.7% (20.7, 50.6) | 5.2% (-17.3, 18.1) | 1.16 (0.48, 1.64) |

*The risk difference for this row is the adjusted risk-difference and risk ratio

For parametric models, risks, risk differences, and risk ratio confidence intervals generated through sandwich approach.

OM = outcome model-based estimator using parametric models

IOW = inverse odds weighting estimator with normalized weights using parametric models

DR = doubly robust estimator using parametric models for all nuisance functions

DDML = double debiased machine learner - doubly robust estimator using ensemble learner and cross fitting for all nuisance functions

95% confidence intervals in parentheses

**Table S2 Meis (n=459) transported to PROLONG (n=1684)**

| Estimator | 17P | Placebo | Risk Difference | Risk Ratio |
| --- | --- | --- | --- | --- |
| PROLONG* | 22.9% | 21.9% | 1.0% (-3.1, 4.8) | 1.04 (0.87, 1.26) |
| OM | 39.9% (30.2, 51.0) | 55.1% (35.7, 72.8) | -15.2% (-35.0, 6.6) | 0.72 (0.49, 1.18) |
| IOW | 37.3% (26.3, 49.9) | 63.2% (44.6, 76.6) | -25.8% (-43.4, -4.5) | 0.59 (0.40, 0.91) |
| DR | 37.6 (27.1, 50.0) | 63.6 (42.9, 80.3) | -26.0 (-46.2, -2.9) | 0.59 (0.39, 0.94) |
| DDML | 39.1% (20.8, 55.2) | 57.7% (33.9, 89.1) | -18.6% (-55.9, 8.8) | 0.68 (0.31, 1.24) |

*The risk difference for this row is the adjusted risk-difference and risk ratio

OM = outcome model-based estimator using parametric models

IOW = inverse odds weighting estimator with normalized weights using parametric models

DR = doubly robust estimator using parametric models for all nuisance functions

DDML = double debiased machine learner - doubly robust estimator using ensemble learner and cross fitting for all nuisance functions

95% confidence intervals in parentheses

**Table S3 PROLONG-US (n=387) transported to Meis (n=459) (excluding all PROLONG participants not in US)**

| Estimator | 17P | Placebo | Risk Difference | Risk Ratio |
| --- | --- | --- | --- | --- |
| Meis* | 36.8% | 54.3% | -17.5% (-27.7, -8.2) | 0.68 (0.55, 0.84) |
| OM | 41.5% (33.3, 50.8) | 39.1% (27.8, 51.8) | 2.4% (-12.5, 17.0) | 1.06 (0.73, 1.59) |
| IOW | 40.6% (30.8, 50.5) | 34.1% (23.0, 47.9) | 6.5% (-10.9, 21.1) | 1.19 (0.76, 1.86) |
| DR | 42.4% (33.4, 51.9) | 38.1% (26.0, 51.3) | 4.4% (-11.7, 19.4) | 1.11 (0.75, 1.71) |
| DDML | 39.7% (22.2, 48.2) | 34.8% (18.7, 57.0) | 4.9% (-25.3, 20.0) | 1.14 (0.62, 1.73) |

*The risk difference for this row is the adjusted risk-difference and risk ratio

OM = outcome model-based estimator using parametric models

IOW = inverse odds weighting estimator with normalized weights using parametric models

DR = doubly robust estimator using parametric models for all nuisance functions

DDML = double debiased machine learner - doubly robust estimator using ensemble learner and cross fitting for all nuisance functions

95% confidence intervals in parentheses

**Table S4 Meis (n=459) transported to PROLONG-US (n=387) (excluding PROLONG participants not in US)**

| Estimator | 17P | Placebo | Risk Difference | Risk Ratio |
| --- | --- | --- | --- | --- |
| PROLONG* | 22.9% | 21.9% | 1.0% (-3.1, 4.8) | 1.04 (0.87, 1.26) |
| PROLONG-US* | 33.2% | 27.5% | 5.8% (-5.0, 14.8) | 1.21 (0.85, 1.68) |
| OM | 38.4% (31.0, 46.6) | 49.0% (37.6, 60.6) | -10.6% (-24.7, 3.5) | 0.78 (0.58, 1.09) |
| IOW | 36.8% (28.9, 46.0) | 53.0% (39.4, 65.2) | -16.2% (-30.7, 0.3) | 0.69 (0.50, 1.01) |
| DR | 37.2% (29.6, 46.2) | 51.6% (40.4, 64.1) | -14.4% (-29.4, -0.3) | 0.72 (0.52, 1.00) |
| DDML | 33.3% (23.9, 45.5) | 48.0% (32.9, 68.4) | -14.6% (-36.7, 4.8) | 0.69 (0.42, 1.12) |

*The risk difference for this row is the adjusted risk-difference and risk ratio

PROLONG-US = estimates for trial participants in PROLONG from the US

OM = outcome model-based estimator using parametric models

IOW = inverse odds weighting estimator with normalized weights using parametric models

DR = doubly robust estimator using parametric models for all nuisance functions

DDML = double debiased machine learner - doubly robust estimator using ensemble learner and cross fitting for all nuisance functions

95% confidence intervals in parentheses

**Table S5 PROLONG (n=1468) transported to Meis (n=310) (among individuals with only 1 prior preterm delivery)**

| Estimator | 17P | Placebo | Risk Difference | Risk Ratio |
| --- | --- | --- | --- | --- |
| Meis* | 36.8% | 54.3% | -17.5% (-27.7, -8.2) | 0.68 (0.55, 0.84) |
| Meis-1PPTB* | 32.1% | 46.7% | -14.5% (-27.0, -1.7) | 0.69 (0.51, 0.95) |
| OM | - | - | - | - |
| IOW | - | - | - | - |
| DR | - | - | - | - |
| DDML | 34.2% (22.5, 44.9) | 31.5% (13.6, 46.7) | 2.7% (-16.3, 23.4) | 1.09 (0.58, 2.26) |

*The risk difference for this row is the adjusted risk-difference and risk ratio

- denotes missing values because of convergence issues due to insufficient sample size

Meis-1PPTB = estimates for trial participants in Meis with only one prior PTB

OM = outcome model-based estimator using parametric models

IOW = inverse odds weighting estimator with normalized weights using parametric models

DR = doubly robust estimator using parametric models for all nuisance functions

DDML = double debiased machine learner - doubly robust estimator using ensemble learner and cross fitting for all nuisance functions

95% confidence intervals in parentheses

**Table S6 Meis (n=310) transported to PROLONG (n=1468) (among individuals with only 1 prior preterm delivery)**

| Estimator | 17P | Placebo | Risk Difference | Risk Ratio |
| --- | --- | --- | --- | --- |
| PROLONG* | 23.1% | 21.9% | 1.1% (-3.3, 5.2) | 1.04 (0.87, 1.26) |
| PROLONG-1PPTB* | 19.9% | 20.0% | 0% (-4.5, 4.2) | 0.99 (0.80, 1.24) |
| OM | - | - | - | - |
| IOW | - | - | - | - |
| DR | - | - | - | - |
| DDML | - | - | - | - |

*The risk difference for this row is the adjusted risk-difference and risk ratio

- denotes missing values due to insufficient sample size

PROLONG-1PPTB = estimates for trial participants in PROLONG with only one prior PTB

OM = outcome model-based estimator using parametric models

IOW = inverse odds weighting estimator with normalized weights using parametric models

DR = doubly robust estimator using parametric models for all nuisance functions

DDML = double debiased machine learner - doubly robust estimator using ensemble learner and cross fitting for all nuisance functions

95% confidence intervals in parentheses

**Table S7 PROLONG (n=1682) transported to Meis (n=459) (restrict where P(PROLONG|X)>.1)**

| Estimator | 17P | Placebo | Risk Difference | Risk Ratio |
| --- | --- | --- | --- | --- |
| Meis* | 36.8% | 54.3% | -17.5% (-27.7, -8.2) | 0.68 (0.55, 0.84) |
| OM | 35.2% (27.8, 43.2) | 34.5% (24.5, 45.5) | 0.7% (-12.6, 13.1) | 1.02 (0.70, 1.50) |
| IOW | 33.7% (24.6, 45.3) | 36.4% (23.4, 52.1) | -2.8% (-20.4, 14.3) | 0.92 (0.57, 1.58) |
| DR | 34.6% (26.5, 43.5) | 35.2% (22.8, 48.0) | -0.6% (-15.1, 14.8) | 0.98 (0.66, 1.63) |
| DDML | 34.9% (24.9, 43.1) | 33.7% (20.5, 50.5) | 1.2% (-19.0, 15.9) | 1.04 (0.58, 1.62) |

*The risk difference for this row is the adjusted risk-difference and risk ratio

OM = outcome model-based estimator using parametric models

IOW = inverse odds weighting estimator with normalized weights using parametric models

DR = doubly robust estimator using parametric models for all nuisance functions

DDML = double debiased machine learner - doubly robust estimator using ensemble learner and cross fitting for all nuisance functions

95% confidence intervals in parentheses

**Table S8 Meis (n=434) transported to PROLONG (n=1684) (restrict where P(Meis|X)>.1)**

| Estimator | 17P | Placebo | Risk Difference | Risk Ratio |
| --- | --- | --- | --- | --- |
| PROLONG* | 22.9% | 21.9% | 1.0% (-3.1, 4.8) | 1.04 (0.87, 1.26) |
| OM | - | - | - | - |
| IOW | - | - | - | - |
| DR | - | - | - | - |
| DDML | 35.2% (10.5, 57.0) | 54.3% (20.3, 88.6) | -19.2% (-61.9, 20.6) | 0.61 (0.20, 1.87) |

*The risk difference for this row is the adjusted risk-difference and risk ratio

- denotes missing values because of convergence issues due to insufficient sample size

OM = outcome model-based estimator using parametric models

IOW = inverse odds weighting estimator with normalized weights using parametric models

DR = doubly robust estimator using parametric models for all nuisance functions

DDML = double debiased machine learner - doubly robust estimator using ensemble learner and cross fitting for all nuisance functions

95% confidence intervals in parentheses

**Table S9 PROLONG (n=1528) transported to Meis (n=338) (among individuals who did not smoke, drink alcohol, or use substances during pregnancy)**

| Estimator | 17P | Placebo | Risk Difference | Risk Ratio |
| --- | --- | --- | --- | --- |
| Meis* | 36.8% | 54.3% | -17.5% (-27.7, -8.2) | 0.68 (0.55, 0.84) |
| Meis-no use* | 37.8% | 51.3% | -13.5% (-25.1, -2.2) | 0.74 (0.57, 0.95) |
| OM | 36.6% (27.5, 47.3) | 36.6% (24.9, 51.2) | 0% (-16.8, 15.7) | 1.00 (0.63, 1.61) |
| IOW | 38.1% (26.3, 57.2) | 34.1% (21.8, 49.5) | 4.0% (-14.7, 26.3) | 1.12 (0.65, 2.12) |
| DR | 36.4% (26.6, 49.1) | 38.3% (25.5, 54.7) | -1.9% (-20.2, 16.1) | 0.95 (0.58, 1.62) |
| DDML | 35.6% (21.1, 46.6) | 32.4% (14.2, 51.9) | 3.3% (-21.8, 23.5) | 1.10 (0.51, 2.19) |

*The risk difference for this row is the adjusted risk-difference and risk ratio

Meis-no use = estimates for trial participants in Meis who did not smoke, drink alcohol, or use substances during pregnancy

OM = outcome model-based estimator using parametric models

IOW = inverse odds weighting estimator with normalized weights using parametric models

DR = doubly robust estimator using parametric models for all nuisance functions

DDML = double debiased machine learner - doubly robust estimator using ensemble learner and cross fitting for all nuisance functions

95% confidence intervals in parentheses

**Table S10 Meis (n=338) transported to PROLONG (n=1528) (among individuals who did not smoke, drink alcohol, or use substances during pregnancy)**

| Estimator | 17P | Placebo | Risk Difference | Risk Ratio |
| --- | --- | --- | --- | --- |
| PROLONG* | 22.9% | 21.9% | 1.0% (-3.1, 4.8) | 1.04 (0.87, 1.26) |
| PROLONG-no use* | 22.1% | 20.5% | 1.6% (-2.8, 5.7) | 1.08 (0.88, 1.32) |
| OM | 39.7% (28.3, 53.3) | 50.1% (23.3, 72.8) | -10.4 (-35.8, 19.5) | 0.79 (0.47, 1.79) |
| IOW | 39.9% (26.7, 56.1) | 64.0% (38.4, 79.9) | -23.2 (-45.1, 5.2) | 0.62 (0.39, 1.11) |
| DR | 39.3% (27.3, 55.2) | 64.1% (36.0, 83.6) | -24.8 (-47.8, 7.0) | 0.61 (0.38, 1.19) |
| DDML | - | - | - | - |

*The risk difference for this row is the adjusted risk-difference and risk ratio

PROLONG-no use = estimates for trial participants in PROLONG who did not smoke, drink alcohol, or use substances during pregnancy

- denotes missing values due to insufficient sample size

OM = outcome model-based estimator using parametric models

IOW = inverse odds weighting estimator with normalized weights using parametric models

DR = doubly robust estimator using parametric models for all nuisance functions

DDML = double debiased machine learner - doubly robust estimator using ensemble learner and cross fitting for all nuisance functions

95% confidence intervals in parentheses

**Table S11 PROLONG (n=156) transported to Meis (n=121) (among individuals who did smoke, drink alcohol, or use substances during pregnancy)**

| Estimator | 17P | Placebo | Risk Difference | Risk Ratio |
| --- | --- | --- | --- | --- |
| Meis* | 36.8% | 54.3% | -17.5% (-27.7, -8.2) | 0.68 (0.55, 0.84) |
| Meis-no use* | 37.8% | 51.3% | -13.5% (-25.1, -2.2) | 0.74 (0.57, 0.95) |
| OM | 39.5% (26.0, 51.7) | 38.6% (18.9, 57.7) | 0.8% (-21.7, 24.6) | 1.02 (0.59, 2.06) |
| IOW | 39.9% (25.2, 56.7) | 31.9% (9.3, 68.1) | 8.0% (-29.0, 35.8) | 1.25 (0.51, 4.19) |
| DR | 41.3% (26.9, 55.3) | 34.7% (10.1, 53.8) | 6.6% (-16.3, 35.8) | 1.19 (0.69, 3.66) |
| DDML | - | - | - | - |

*The risk difference for this row is the adjusted risk-difference and risk ratio

Meis-no use = estimates for trial participants in Meis who did smoke, drink alcohol, or use substances during pregnancy

- denotes missing values due to insufficient sample size

OM = outcome model-based estimator using parametric models

IOW = inverse odds weighting estimator with normalized weights using parametric models

DR = doubly robust estimator using parametric models for all nuisance functions

DDML = double debiased machine learner - doubly robust estimator using ensemble learner and cross fitting for all nuisance functions

95% confidence intervals in parentheses

**Table S12 Meis (n=121) transported to PROLONG (n=156) (among individuals who did smoke, drink alcohol, or use substances during pregnancy)**

| Estimator | 17P | Placebo | Risk Difference | Risk Ratio |
| --- | --- | --- | --- | --- |
| PROLONG* | 22.9% | 21.9% | 1.0% (-3.1, 4.8) | 1.04 (0.87, 1.26) |
| PROLONG-no use* | 32.8% | 34.2% | -1.4% (-2.2, 16.4) | 0.96 (0.55, 1.80) |
| OM | 30.9% (18.5, 45.4) | 67.9% (45.7, 83.9) | -36.9% (-58.9, -12.1) | 0.46 (0.24, 0.77) |
| IOW | 29.4% (16.9, 48.4) | 69.4% (38.1, 88.6) | -40.0% (-64.0, -6.9) | 0.42 (0.22, 0.84) |
| DR | 30.5% (17.6, 48.4) | 68.7% (49.3, 85.2) | -38.2% (-61.5, -13.6) | 0.44 (0.22, 0.79) |
| DDML | - | - | - | - |

*The risk difference for this row is the adjusted risk-difference and risk ratio

PROLONG-no use = estimates for trial participants in PROLONG who did smoke, drink alcohol, or use substances during pregnancy

- denotes missing values due to insufficient sample size

OM = outcome model-based estimator using parametric models

IOW = inverse odds weighting estimator with normalized weights using parametric models

DR = doubly robust estimator using parametric models for all nuisance functions

DDML = double debiased machine learner - doubly robust estimator using ensemble learner and cross fitting for all nuisance functions

95% confidence intervals in parentheses

**Table S13 PROLONG (n=587) transported to Meis (n=106) (among individuals had a previous preterm premature rupture of membranes delivery)**

| Estimator | 17P | Placebo | Risk Difference | Risk Ratio |
| --- | --- | --- | --- | --- |
| Meis* | 36.8% | 54.3% | -17.5% (-27.7, -8.2) | 0.68 (0.55, 0.84) |
| Meis-PPROM* | 42.9% | 52.3% | -9.3% (-33.7, 14.6) | 0.82 (0.50, 1.45) |
| OM | 29.6% (20.5, 42.4) | 34.2% (18.7, 52.7) | -4.6% (-24.2, 15.6) | 0.87 (0.49, 1.80) |
| IOW | 27.9% (18.3, 43.6) | 35.1% (13.7, 56.0) | -7.2% (-29.9, 19.8) | 0.79 (0.42, 2.19) |
| DR | 28.8% (20.1, 42.5) | 35.9% (16.1, 54.5) | -7.0 (-26.7, 18.7) | 0.80 (0.46, 2.25) |
| DDML | 34.9% (19.9, 44.2) | 32.1% (13.2, 59.2) | 2.9% (-30.0, 21.8) | 1.09 (0.41, 2.08) |

*The risk difference for this row is the adjusted risk-difference and risk ratio

Meis-PPROM = estimates for trial participants in Meis who had a previous preterm premature rupture of membranes delivery

OM = outcome model-based estimator using parametric models

IOW = inverse odds weighting estimator with normalized weights using parametric models

DR = doubly robust estimator using parametric models for all nuisance functions

DDML = double debiased machine learner - doubly robust estimator using ensemble learner and cross fitting for all nuisance functions

95% confidence intervals in parentheses

**Table S14 Meis (n=106) transported to PROLONG (n=587) (among individuals had a previous preterm premature rupture of membranes delivery)**

| Estimator | 17P | Placebo | Risk Difference | Risk Ratio |
| --- | --- | --- | --- | --- |
| PROLONG* | 22.9% | 21.9% | 1.0% (-3.1, 4.8) | 1.04 (0.87, 1.26) |
| PROLONG- PPROM* | 27.0% | 25.7% | 1.4% (-6.5, 8.9) | 1.05 (0.79, 1.43) |
| OM | 47.8% (28.8, 65.2) | 57.7% (25.9, 85.8) | -9.9 (-45.2, 25.5) | 0.83 (0.41, 1.84) |
| IOW | 37.1% (15.8, 67.2) | 72.0% (30.2, 93.5) | -34.9 (-68.6, 11.8) | 0.52 (0.18, 1.30) |
| DR | 44.5% (24.6, 63.6) | 67.9% (30.6, 92.9) | -23.4 (-60.0, 15.1) | 0.66 (0.27, 1.41) |
| DDML | - | - | - | - |

*The risk difference for this row is the adjusted risk-difference and risk ratio

PROLONG-PPROM = estimates for trial participants in PROLONG who had a previous preterm premature rupture of membranes delivery

- denotes missing values due to insufficient sample size

OM = outcome model-based estimator using parametric models

IOW = inverse odds weighting estimator with normalized weights using parametric models

DR = doubly robust estimator using parametric models for all nuisance functions

DDML = double debiased machine learner - doubly robust estimator using ensemble learner and cross fitting for all nuisance functions

95% confidence intervals in parentheses

**Table S15 PROLONG (n=1095) transported to Meis (n=353) (among individuals who did not have a previous preterm premature rupture of membranes delivery)**

| Estimator | 17P | Placebo | Risk Difference | Risk Ratio |
| --- | --- | --- | --- | --- |
| Meis* | 36.8% | 54.3% | -17.5% (-27.7, -8.2) | 0.68 (0.55, 0.84) |
| Meis-no PPROM* | 34.9% | 55.9% | -21.0% (-31.9, -9.9) | 0.62 (0.49, 0.79) |
| OM | 44.1% (32.6, 54.1) | 32.3% (20.8, 47.7) | 11.8% (-7.2, 27.1) | 1.37 (0.83, 2.20) |
| IOW | 47.1% (26.0, 66.0) | 23.0% (12.2, 38.4) | 24.1% (-0.8, 46.9) | 2.04 (0.97, 4.21) |
| DR | 45.3% (27.2, 59.2) | 24.6% (12.2, 42.5) | 20.7% (-3.5, 40.8) | 1.84 (0.93, 3.87) |
| DDML | 41.7% (29.2, 55.8) | 30.6% (15.2, 47.4) | 11.0% (-9.7, 32.1) | 1.36 (0.73, 2.64) |

*The risk difference for this row is the adjusted risk-difference and risk ratio

Meis-no PPROM = estimates for trial participants in Meis who did not have a previous preterm premature rupture of membranes delivery

OM = outcome model-based estimator using parametric models

IOW = inverse odds weighting estimator with normalized weights using parametric models

DR = doubly robust estimator using parametric models for all nuisance functions

DDML = double debiased machine learner - doubly robust estimator using ensemble learner and cross fitting for all nuisance functions

95% confidence intervals in parentheses

**Table S16 Meis (n=353) transported to PROLONG (n=1095) (among individuals who did not have a previous preterm premature rupture of membranes delivery)**

| Estimator | 17P | Placebo | Risk Difference | Risk Ratio |
| --- | --- | --- | --- | --- |
| PROLONG* | 22.9% | 21.9% | 1.0% (-3.1, 4.8) | 1.04 (0.87, 1.26) |
| PROLONG-no PPROM* | 21.0% | 20.2% | 0% (-4.4, 5.6) | 1.04 (0.81, 1.34) |
| OM | 38.1% (28.4, 52.7) | 53.4% (26.7, 75.0) | -15.3% (-38.8, 14.2) | 0.71 (0.45, 1.58) |
| IOW | 37.7% (26.4, 56.2) | 63.3% (39.2, 80.9) | -25.6% (-46.5, 5.2) | 0.60 (0.38, 1.20) |
| DR | 33.7% (22.7, 51.2) | 66.3% (39.3, 88.8) | -32.5% (-57.4, -0.2) | 0.51 (0.30, 1.05) |
| DDML | 22.7% (9.1, 47.4) | 68.4% (38.2, 99.9) | -45.8% (-76.2, -5.4) | 0.33 (0.14, 1.08) |

*The risk difference for this row is the adjusted risk-difference and risk ratio

PROLONG-no PPROM = estimates for trial participants in PROLONG who did not have a previous preterm premature rupture of membranes delivery

OM = outcome model-based estimator using parametric models

IOW = inverse odds weighting estimator with normalized weights using parametric models

DR = doubly robust estimator using parametric models for all nuisance functions

DDML = double debiased machine learner - doubly robust estimator using ensemble learner and cross fitting for all nuisance functions

95% confidence intervals in parentheses

**Table S17 PROLONG (n=1684) transported to Meis (n=459) for outcome of spontaneous PTB**

| Estimator | 17P | Placebo | Risk Difference | Risk Ratio |
| --- | --- | --- | --- | --- |
| Meis* | 29.8% | 43.3% | -13.5% (-23.1, -4.5) | 0.69 (0.53, 0.88) |
| OM | 29.0% (22.1, 36.9) | 24.1% (15.8, 35.3) | 4.9% (-7.6, 16.4) | 1.20 (0.76, 2.00) |
| IOW | 29.6% (20.2, 42.3) | 24.1% (14.5, 38.9) | 5.4% (-11.2, 21.3) | 1.23 (0.65, 2.36) |
| DR | 30.7% (22.3, 41.4) | 26.1% (16.2, 40.4) | 4.6% (-11.1, 19.2) | 1.17 (0.68, 2.16) |
| DDML | 29.1% (20.2, 38.9) | 23.8% (11.5, 39.3) | 5.3% (-12.6, 20.8) | 1.22 (0.62, 2.31) |

*The risk difference for this row is the adjusted risk-difference and risk ratio

For parametric models, risks, risk differences, and risk ratio confidence intervals generated through sandwich approach.

OM = outcome model-based estimator using parametric models

IOW = inverse odds weighting estimator with normalized weights using parametric models

DR = doubly robust estimator using parametric models for all nuisance functions

DDML = double debiased machine learner - doubly robust estimator using ensemble learner and cross fitting for all nuisance functions

95% confidence intervals in parentheses

**Table S18 Meis (n=459) transported to PROLONG (n=1684) for outcome of spontaneous PTB**

| Estimator | 17P | Placebo | Risk Difference | Risk Ratio |
| --- | --- | --- | --- | --- |
| PROLONG* | 18.6% | 17.0% | 1.6% (-2.4, 5.3) | 1.09 (0.88, 1.4) |
| OM | 31.9% (22.9, 43.0) | 35.7% (21.7, 54.1) | -3.8% (-23.8, 13.3) | 0.89 (0.51, 1.59) |
| IOW | 31.8% (21.2, 44.3) | 43.2% (26.9, 60.1) | -11.4% (-31.5, 8.8) | 0.74 (0.43, 1.30) |
| DR | 31.9% (22.1, 44.5) | 40.9% (25.1, 58.7) | -8.9% (-29.6, 10.3) | 0.78 (0.45, 1.40) |
| DDML | 32.1% (13.3, 46.8) | 35.7% (14.0, 67.9) | -3.6% (-42.5, 20.7) | 0.90 (0.27, 2.08) |

*The risk difference for this row is the adjusted risk-difference and risk ratio

OM = outcome model-based estimator using parametric models

IOW = inverse odds weighting estimator with normalized weights using parametric models

DR = doubly robust estimator using parametric models for all nuisance functions

DDML = double debiased machine learner - doubly robust estimator using ensemble learner and cross fitting for all nuisance functions

95% confidence intervals in parentheses

**Table S19 PROLONG (n=1684) transported to Meis (n=459) for outcome of preterm premature rupture of membranes delivery**

| Estimator | 17P | Placebo | Risk Difference | Risk Ratio |
| --- | --- | --- | --- | --- |
| Meis* | 7.0% | 9.8% | -2.8% (-8.8, 2.3) | 0.71 (0.37, 1.40) |
| OM | 7.8% (4.2, 13.7) | 6.3% (1.8, 15.1) | 1.5% (-7.3, 8.0) | 1.23 (0.39, 4.91) |
| IOW | 7.1% (3.5, 15.9) | 7.4% (2.2, 22.7) | -0.2% (-13.6, 7.1) | 0.97 (0.22, 4.20) |
| DR | 8.5% (4.4, 16.3) | 8.8% (2.4, 19.7) | -0.2% (-11.5, 8.3) | 0.98 (0.29, 4.57) |
| DDML | 6.0% (2.7, 12.8) | 7.9% (-2.7, 22.8) | -1.9% (-16.1, 11.5) | 0.75 (0.17, 4.73) |

*The risk difference for this row is the adjusted risk-difference and risk ratio

For parametric models, risks, risk differences, and risk ratio confidence intervals generated through sandwich approach.

OM = outcome model-based estimator using parametric models

IOW = inverse odds weighting estimator with normalized weights using parametric models

DR = doubly robust estimator using parametric models for all nuisance functions

DDML = double debiased machine learner - doubly robust estimator using ensemble learner and cross fitting for all nuisance functions

95% confidence intervals in parentheses

**Table S20 Meis (n=459) transported to PROLONG (n=1684) for outcome of preterm premature rupture of membranes delivery**

| Estimator | 17P | Placebo | Risk Difference | Risk Ratio |
| --- | --- | --- | --- | --- |
| PROLONG* | 5.6% | 3.6% | 2.0% (0, 3.9) | 1.54 (0.96, 2.58) |
| OM | 4.2% (1.6, 9.9) | 3.1% (1.1, 5.8) | 1.1% (-2.4, 7.0) | 1.37 (0.39, 5.42) |
| IOW | 5.4% (1.7, 14.6) | 4.2% (1.7, 10.1) | 1.2% (-4.3, 9.0) | 1.28 (0.21, 5.51) |
| DR | 4.9% (1.6, 12.6) | 3.0% (0.7, 5.8) | 2.0% (-2.1, 10.2) | 1.66 (0.47, 9.34) |
| DDML | 2.7% (0.5, 8.4) | 4.2% (0.9, 12.2) | -1.4% (-14.3, 10.9) | 0.65 (0.23, 8.9) |

*The risk difference for this row is the adjusted risk-difference and risk ratio

OM = outcome model-based estimator using parametric models

IOW = inverse odds weighting estimator with normalized weights using parametric models

DR = doubly robust estimator using parametric models for all nuisance functions

DDML = double debiased machine learner - doubly robust estimator using ensemble learner and cross fitting for all nuisance functions

95% confidence intervals in parentheses

Appendix S3. Outcome distribution for delivery during trial

|  | PROLONG n=1681* | Meis  N=459 |
| --- | --- | --- |
| Preterm delivery for fetal indication | 29 | 13 |
| Preterm delivery for maternal indication | 43 | 23 |
| PROM leading to preterm induction / Cesarean section | 35 | 19 |
| PROM leading to spontaneous preterm delivery | 48 | 19 |
| Spontaneous loss (<20 weeks age of gestation) | 10 | 5 |
| Spontaneous preterm labor preceded delivery | 214 | 116 |
| Term (gestational age >= 37 weeks) | 1302 | 264 |

*3 missing classification from PROLONG

1.	Burton, A. & Altman, D.G. Missing covariate data within cancer prognostic studies: a review of current reporting and proposed guidelines. *Br J Cancer* **91**, 4-8 (2004).

2.	Miettinen, O.S. *Theoretical epidemiology : principles of occurrence research in medicine*, (Wiley, New York, 1985).
